# Efficacy and safety of Reyanning mixture combined with conventional Western medicine for treating COVID-19: A protocol for systematic review and meta-analysis

**DOI:** 10.1097/MD.0000000000024169

**Published:** 2021-01-22

**Authors:** Yajuan Li, Jingxia Zhang, Shasha Li, Fan Li, Chongbo Zhao, Fang Li, Weifeng Wang, Wei Wang

**Affiliations:** a Pharmacy College, Shaanxi University of Chinese Medicine, Xianyang; b Shaanxi Academy of Traditional Chinese Medicine, Xi’an, Shaanxi, China.

**Keywords:** Conventional western medicine, corona virus disease 2019, meta-analysis, Reyanning mixture

## Abstract

**Background:**

Since its first report in December 2019, coronavirus disease 2019 (COVID-19), caused by severe acute respiratory syndrome coronavirus 2 (SARS-CoV-2), has rapidly emerged as a pandemic affecting nearly all countries worldwide. So far, there is no specific anti-coronavirus therapy approved for the treatment of COVID-19. In China, some traditional Chinese medicines (TCM) have been successfully applied to the treatment of SARS-CoV-2 and have achieved good clinical results, including the Reyanning mixture, but there is no systematic review about it. This study will systematically evaluate its efficacy and safety in the treatment of COVID-19.

**Methods:**

The following electronic bibliographic databases will be searched to identify relevant studies: PubMed, MEDLINE, EMBASE, CNKI, CBM, and Wanfang databases. We will use the Cochrane Handbook for Systematic Reviews of Interventions to assess the risk of bias. The protocol will be conducted according to the approach and Preferred Reporting Items for Systematic Review and Meta-Analysis Protocols (PRISMA-P). Manager 5.3 software and STATA 16.0 software were used to perform the meta-analysis.

**Results:**

The systematic review and meta-analysis aims to review and pool current clinical outcomes of Reyanning mixture for the treatment of COVID-19.

**Conclusion:**

The conclusion of this review will provide evidence to judge whether Reyanning mixture combined with Conventional Western Medicine is an effective and safe intervention for COVID-19.

**INPLASY registration number:**

INPLASY2020120044.

## 1 Introduction

Coronavirus disease 2019 (COVID-19) is a global pandemic caused by the Severe Acute Respiratory Syndrome Coronavirus-2 (SARS-CoV-2).^[1]^ It has become a key issue that has seriously threatened the public health of people around the world.^[2]^

According to statistics from the World Health Organization, as of December 7, 2020, there were 66.2 million confirmed cases worldwide, with a total of 1.5 million deaths.^[3]^ In China, the coronavirus disease 2019 (COVID-19) has been classified as class B infectious diseases (class a management).^[4]^ Clinicians mainly use symptomatic support therapy since there is currently no specific medicine that can be used to cure the disease.

Recently, as the efficacy and safety of traditional Chinese medicine (TCM) is widely acknowledged, it has been brought to a crucial status by the public, governments, and World Health Organization (WHO). Some studies have shown that the combination of Chinese and Western medicine can achieve some effect on the treatment of COVID-19.^[5–7]^ Reyanning mixture was recommended as the treatment agent of the “Diagnosis and Treatment Scheme for New Coronavirus Infected Pneumonia of Shaanxi Province (Trial Version 2).”^[8]^ Reyanning mixture is a kind of Chinese pharmacopeia. It is composed of 4 herbs: Taraxacum officinalis, family Asteraceae (PU GONG YING); Polygonum cuspidatum, family Polygonaceae (HU ZHANG); Patrinia villosa, family Valerianaceae (BAI JIANG CAO); and Scutellaria barbata, family Lamiaceae (BAN ZHI LIAN). It has the function of clearing away heat and detoxification. Clinical statistics show that Reyanning mixture can improve the clinical symptoms of COVID-19 patients, promote the improvement of chest CT, shorten the patient's fever time, and increase the rate of nucleic acid conversion.^[9]^ But its efficacy lacks evidence-based medical evaluation. Thus, we propose a protocol of systematic review to evaluate the efficacy and safety of Reyanning mixture combined with CWM vs CWM for COVID-19.

## 2 Methods and program

Our protocol has been registered on the International Platform of Registered Systematic Review and Meta-Analysis Protocols (INPLASY). The registration number was INPLASY2020120044. We strictly abide by Preferred Reporting Items for Systematic Review and Meta-Analysis Protocols (PRISMA-P) guidelines.^[10]^

### 2.1 Data sources and retrieval strategy

Studies were obtained from PubMed, MEDLINE, EMBASE, the China National Knowledge Infrastructure (CNKI), China Biomedical Database (CBM), Wan Fang databases, regardless of publication date or language. The databases were searched by combining the subject words with random words. The retrieval strategy is shown in Table 1 using PubMed retrieval as an example. The search terms were adapted appropriately to conform to different syntax rules of different databases.

**Table 1:** PubMed search strategy.

Number	Term
1	“COVID-19” [MeSH] OR “2019 novel coronavirus disease” [Title/Abstract]OR “SARS-CoV-2 infection” [Title/Abstract] OR “COVID-19 virus disease”[Title/Abstract]OR “COVID-19 pandemic” [Title/Abstract] OR “2019 novel coronavirusinfection” [Title/Abstract]OR “COVID19” [Title/Abstract] OR “2019-nCoV disease” [Title/Abstract]OR “COVID-19 virus infection” [Title/Abstract] OR “2019-nCoV infection”[Title/Abstract]OR “coronavirus disease 2019” [Title/Abstract] OR “coronavirus disease-19” [Title/Abstract]
2	“Reyanning mixture” [Title/Abstract] OR “Reyanning Formula” [Title/Abstract]
3	“randomized controlled trial” [Title/Abstract] OR “controlled clinical trial” [Title/Abstract] OR “Single-Blind Method” [Text Word] OR “random allocation” [Text Word] OR “RCT” [Text Word] OR “RCTs” [Text Word]
4	1 AND 2 AND 3

### 2.2 Inclusion criteria

#### 2.2.1 Study design

The study only select clinical randomized controlled trials of Reyanning mixture for COVID-19 published in both Chinese and English. However, animal experiments, reviews, case reports, and non-randomized controlled trials are excluded.

#### 2.2.2 Participants

This study included common-type patients who had been clearly diagnosed with the new coronavirus disease. Except that participants must be between 18 and 80 years old, there are no strict gender restrictions.

#### 2.2.3 Intervention group

The test group uses Reyanning mixture on the basis of conventional drug treatment. The control group can be treated with only conventional drug.

#### 2.2.4 Outcomes

Total clinical effective rate, the symptom disappearance rates (throat dryness, throat pain, cough, fever, fatigue, chest tightness, runny nose, nasal congestion, and headache), time to complete fever clearance (d), the nucleic acid conversion rate and time to recovery on chest CT. Additional outcomes: neutrophils, lymphocyte, C-reactive protein, and adverse events.^[9]^

**Figure 1:**
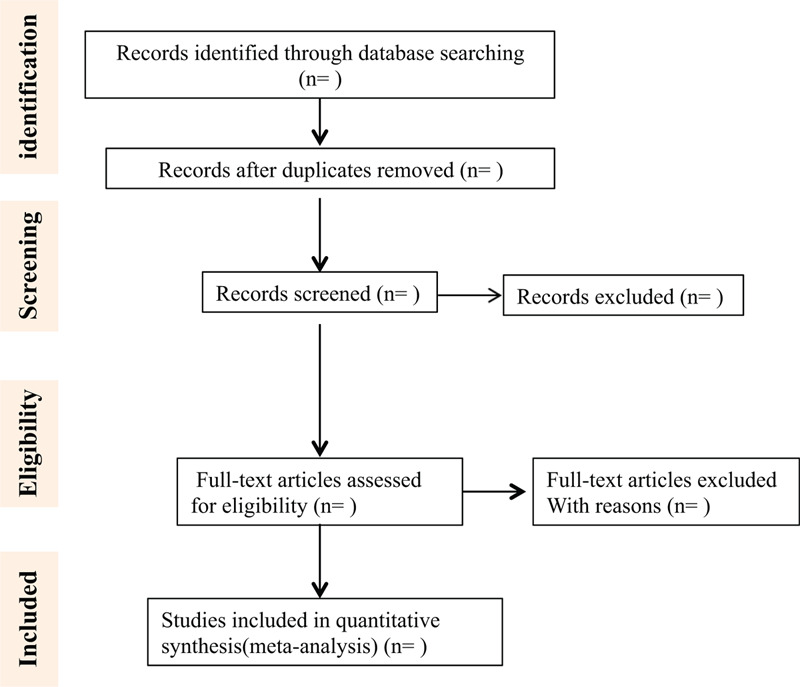
Process of study screening.

The PRISMA flowchart shows the complete screening process (Fig. 1).

### 2.3 Data extraction and management

According to the characteristics of the study, we prepare an Excel data table to collect data before data extraction. The results of the eligibility index were extracted independently and the data were extracted by two reviewers. The main data extracted are as follows: title, author, year, source of funds, sample size, age, gender, duration of disease, intervention measures, outcome indicators, adverse reactions, etc. If there is anything unclear, you can contact the author for more details without hesitation. The above information was finally cross checked by two reviewers.

### 2.4 Assessment of risk of bias in included studies

The quality assessment of RCTs adopts the risk of bias (ROB) assessment tool provided by the Cochrane Handbook. The following seven items, such as random sequence generation, hidden allocation, blindness of participants and personnel, blind evaluation of results, incomplete results data, selective results reporting, and other biases were classified into three grades: low bias, high bias, and unclear bias. By discussing the differences, a consensus will be reached between the two reviewers or seek third-party consultation.

### 2.5 Data analysis

Data analysis will be performed using Stata 16.0 software. Risk ratio (RR) or odds ratio (or) and 95% confidence interval (CI) will be used for dichotomous outcomes, and 95% CI and mean difference (MD) or standardized mean difference (SMD) will be used for continuous results. The number of treatments required will be calculated to explain the results. Cochrane *X*^2^ and *I*^2^ tests will be conducted to evaluate the heterogeneity analysis between studies. If *P* < .05 and *I*^2^ > 50%, then a random effect model will be used. When *P* > .05 and *I*^2^ < 50%, the fixed effect model will be used.^[11]^ Non-reported deviations will be evaluated by funnel plot and eggertest.^[12]^*P* values <.05 indicate publication bias.

## 3 Discussion

In nearly a year, the worldwide spread of COVID-19 has become a far-reaching threat to human health. In China, TCM is widely used as a treatment option in national and provincial guidelines. The model of TCM and the theory and practice of epidemic prevention and control for thousands of years have laid a solid foundation for the effective prevention and control of COVID-19. So far, the prevention and control of the pandemic situation in China has achieved a phased victory, successfully containing the spread of the virus. From the actual application effect of Hubei and even the whole country, the integrated treatment of traditional Chinese and Western medicine played an important role in epidemic prevention and control, and achieved good results.^[13–15]^

Reyanning mixture, a Chinese patent medicine for clearing away heat and detoxification, has a good effect in the treatment of upper respiratory tract infections, colds, fever, acute pharyngitis, pneumonia and many other respiratory diseases, and its anti-virus effect is remarkable.^[16–19]^ And network pharmacology and molecular docking studies of the Reyanning mixture show that it can inhibit inflammation, regulate immune function and reduce lung damage by regulating key targets (such as tumor necrosis factor, interferon gamma, tumor protein P53, C-reactive protein and peroxisome proliferator- activated receptor gamma), so as to achieve the purpose of treating COVID-19. Our study aims to evaluate the effectiveness and safety of Reyanning mixture in the treatment of COVID-19, therefore, we provide a more reliable basis for future clinical decision-making and guidance development.^[20]^

## Author contributions

**Conceptualization:** Yajuan Li.

**Data curation:** Shasha Li, Fan Li.

**Formal analysis:** Chongbo Zhao, Wei Wang.

**Methodology:** Fang Li.

**Software:** Shasha Li, Chongbo Zhao.

**Visualization:** Jingxia Zhang, Shasha Li.

**Writing – original draft:** Fang Li.

**Writing – review & editing:** Weifeng Wang, Wei Wang.
